# Nutritional Aspects of Essential Trace Elements in Oral Health and Disease: An Extensive Review

**DOI:** 10.1155/2016/5464373

**Published:** 2016-06-28

**Authors:** Preeti Tomar Bhattacharya, Satya Ranjan Misra, Mohsina Hussain

**Affiliations:** ^1^Department of Oral Medicine and Radiology, Haldia Institute of Dental Sciences and Research, Haldia, West Bengal 721645, India; ^2^Department of Oral Medicine and Radiology, Institute of Dental Sciences, Bhubaneswar, Orissa 753001, India

## Abstract

Human body requires certain essential elements in small quantities and their absence or excess may result in severe malfunctioning of the body and even death in extreme cases because these essential trace elements directly influence the metabolic and physiologic processes of the organism. Rapid urbanization and economic development have resulted in drastic changes in diets with developing preference towards refined diet and nutritionally deprived junk food. Poor nutrition can lead to reduced immunity, augmented vulnerability to various oral and systemic diseases, impaired physical and mental growth, and reduced efficiency. Diet and nutrition affect oral health in a variety of ways with influence on craniofacial development and growth and maintenance of dental and oral soft tissues. Oral potentially malignant disorders (OPMD) are treated with antioxidants containing essential trace elements like selenium but even increased dietary intake of trace elements like copper could lead to oral submucous fibrosis. The deficiency or excess of other trace elements like iodine, iron, zinc, and so forth has a profound effect on the body and such conditions are often diagnosed through their early oral manifestations. This review appraises the biological functions of significant trace elements and their role in preservation of oral health and progression of various oral diseases.

## 1. Introduction

Appropriate nutrition of all the metabolically active cells and tissues is essential for preserving health of the human body as a whole. Micronutrients, including trace elements, vitamins, and antioxidants, play a vital role in continuously occurring regenerative processes, coping with ongoing oxidative stress in the body tissues, and sustaining ample immunity against pathogens [1, 2]. The manifestations of undernutrition as well overnutrition of micronutrients on the oral health are vast and can result in defects of the dental hard tissues as well as oral mucosa [3, 4].

The word “trace elements” is used for elements existing in natural and perturbed environments in small amounts, with excess bioavailability having a toxic effect on the living organism [5]. Trace elements are chemical micronutrients which are required rather in minute quantity but play a vital role in maintaining integrity of various physiological and metabolic processes occurring within living tissues. The deficiency of any of the trace elements may be apparent as a combination of various clinical manifestations rather than a specific presentation as each trace element is related to many enzyme systems.

Healthy nutritional habits with regular intake of essential vitamins and minerals are of immense significance to general as well as oral health. As there had been limited knowledge among the oral physicians regarding significance of trace elements in human nutrition, the current review focuses on the role of those essential trace elements which have a proven role in maintaining oral health and their implications in various oral diseases and disorders.

## 2. Classifications of Trace Elements

Limited attempts have been made for classifying trace elements solely. The classifications which address the presence of trace elements have been listed.

### 2.1. WHO Classification, 1973 [6]

As per this classification, nineteen trace elements have been divided into three groups:

1. Essential elements: zinc (Zn), copper (Cu), selenium (Se), chromium (Cr), cobalt (Co), iodine (I), manganese (Mn), and molybdenum (Mo).
2. Probably essential elements.
3. Potentially toxic elements.

### 2.2. Frieden's Classification of Elements

In 1981, Frieden proposed a biological classification of trace elements based on their amount in tissues [7]:

1. Essential trace elements: boron, cobalt, copper, iodine, iron, manganese, molybdenum, and zinc.
2. Probably essential trace elements: chromium, fluorine, nickel, selenium, and vanadium.
3. Physically promotive trace elements: bromine, lithium, silicon, tin, and titanium.

### 2.3. Frieden's Categorical Classification of Elements [8]

Twenty-nine types of elements present in the human body have been classified into five major groups as follows:

- Group I: basic components of macromolecules such as carbohydrates, proteins, and lipids. Examples include carbon, hydrogen, oxygen, and nitrogen.
- Group II: nutritionally important minerals also referred to as principal or macroelements. The daily requirement of these macroelements for an adult person is above 100 mg/day. Examples include sodium, potassium, chloride, calcium, phosphorous, magnesium, and sulfur.
- Group III: essential trace elements. The trace elements are also called minor elements. An element is considered a trace element when its requirement per day is below 100 mg. The deficiency of these elements is rare but may prove fatal. Examples include copper, iron, zinc, chromium, cobalt, iodine, molybdenum, and selenium.
- Group IV: additional trace elements. Their role is yet unclear and they may be essential. Examples include cadmium, nickel, silica, tin, vanadium, and aluminum. This group may be equivalent to probably essential trace elements in the WHO classification.
- Group V: these metals are not essential and their functions are not known. They may produce toxicity in excess amounts. Examples include gold, mercury, and lead. This group is equivalent to potentially toxic elements defined in the WHO classification.

## 3. Discussion

An extensive and complex system is functioning inside the human body to manage and maintain the amount of essential trace elements within a normal range. Micronutrients from diet are transported into the blood if there is deficiency, enter cells if cellular levels are not adequate, or are excreted if  blood and cell levels are satisfactory or elevated. Various essential trace elements along with nutritional requirement and dietary sources have been listed in Table 1.

### 3.1. Copper

Copper is the third most abundant trace element with only 75–100 mg of total amount in the human body [9]. Copper is present in almost every tissue of the body and is stored chiefly in the liver along with the brain, heart, kidney, and muscles [10]. Copper is absorbed in the gut and transported to the liver. In human blood, copper is principally distributed between the erythrocytes and in the plasma [11]. It is transported in the form of ceruloplasmin into the plasma where its metabolism is controlled and is excreted in bile [12]. Ceruloplasmin accounts for 90% of the copper content in blood and is responsible for carrying copper to the deficient cells [13]. Copper-zinc metalloenzyme superoxide dismutase contains 60% of the copper in erythrocytes and the remaining 40% is loosely bound to other proteins and amino acids.

#### 3.1.1. Biological Functions

A significant number of metabolic enzymes function properly due to copper [13–16]. The biological functions of copper have been listed [14–16]:

1. The enzyme cytochrome c oxidase, comprising copper and iron, plays a vital role in energy production during aerobic respiration.
2. Copper is also present in superoxide dismutase which detoxifies superoxides by converting them to oxygen and hydrogen peroxide.
3. Copper is also a component of lysyl oxidase which takes part in the synthesis of collagen and elastin. Copper is also essential for maintaining the strength of the skin, hair, blood vessels, and epithelial and connective tissue throughout the body.
4. Cu plays a considerable role in the production of hemoglobin. Ceruloplasmin catalyzes the oxidation of iron which subsequently is necessary to bind to its transport protein, transferrin [12].
5. Melanin production: copper containing enzyme tyrosinase converts tyrosine to melanin.
6. Myelin production: Cu is also necessary for the synthesis of phospholipids found in myelin sheaths in peripheral nerves [13, 16].
7. Copper is also required for the production of the thyroid hormone thyroxine [13].
8. Copper can act as both an antioxidant and a prooxidant. As an antioxidant, Cu scavenges or neutralizes free radicals and may reduce or help prevent some of the damage they cause [16–19]. Copper promotes free radical damage to the tissues when it acts as prooxidant [20].

#### 3.1.2. Role in Oral Health and Diseases

The symptoms of copper deficiency are hypochromic anemia, neutropenia, hypopigmentation of hair and skin, abnormal bone formation with skeletal fragility and osteoporosis, joint pain, lowered immunity, vascular aberrations, and kinky hair [21]:

1. Deficiency of Cu in diet for a prolonged period especially during stages of active growth leads to anemia and defective keratinisation in the oral cavity [22]. The anemic effect is attributed to decreased ferroxidase activity of ceruloplasmin and reduced iron oxidation [5].
2. Infections: lowered immunity can result in various infections of the oral cavity due to accompanied neutropenia [23]. Granulocyte maturation disorder in the bone marrow and vacuolation in neutrophils have been noted [5].
3. Bone abnormalities and pain: bone changes in copper deficiency include a loss of trabecular formation with thinning of the cortex. There may be osteoporosis and occipital horn formation due to functional impairment of copper-requiring enzymes such as ascorbate oxidase and lysyl oxidase in case of copper deficiency [10].
4. Oral lesions: various studies found that the mean serum copper levels were significantly higher in the sera of patients with oral potentially malignant disorders such as oral leukoplakia and oral submucous fibrosis and also malignant tumors such as squamous cell carcinoma. The average intake of copper in India is 2.1–3.9 mg/day whereas, due to areca nut chewing, it is more than 5 mg/day. It has been postulated that copper released from areca nuts while chewing came in direct contact with the oral epithelium and is dissolved in the saliva. Copper is reportedly present in the saliva for as long as 30 minutes. The longer the presence of copper in saliva, the higher the chances of its uptake by the oral epithelium [24]. It has been advocated that copper appears in the blood after 15 mins of ingestion of areca nut and its products [25]. In oral submucous fibrosis patients, the serum levels of Cu gradually increase as the clinical stage of the disease progresses. However, local effect of raised salivary copper may have a more important role to play than the raised serum levels. Other schools of thoughts appraised decrease in the copper serum concentrations due to usage of copper in upregulation of lysyl oxidase leading to excessive cross linkage of collagen [26].
5. Cu is also believed to possess caries promoting property [27].

### 3.2. Zinc

There is 2–4 grams of Zn distributed throughout the human body [28]. Zinc is stored in prostate, parts of the eye, brain, muscle, bones, kidney, and liver [29]. It is the second most abundant transition metal in organisms after iron and it is the only metal which appears in all enzyme classes [28, 30]. In blood plasma, Zn is bound to and transported by albumin (60%) and transferrin (10%) [31]. Since transferrin also transports iron, excessive iron can reduce zinc absorption, and vice versa [32]. The concentration of zinc in blood plasma stays relatively constant regardless of zinc intake.

#### 3.2.1. Biological Functions

Zinc functions in biology are numerous but can be separated into three main categories: catalytic, regulatory, and structural roles. It is required for the catalytic activity of a large number of enzymes [33, 34]. It plays an important role in immune function, wound healing, protein synthesis, DNA synthesis, and cell division [34–36]. Zinc is required for proper sense of taste and smell [37, 38]. It also supports normal growth and development during pregnancy, childhood, and adolescence [39–42]. Allegedly, it also possesses antioxidant properties and thus may play a role in speeding up the healing process after an injury and protecting against accelerated aging [40, 43]. Zinc ions are effective antimicrobial agents even at low concentrations.

#### 3.2.2. Role in Oral Health and Diseases

 The roles of zinc in oral health and diseases are summarized as follows:

1. In the oral cavity, zinc is present naturally in plaque, saliva, and enamel. Zinc is transformed into oral health products to control plaque, reduce malodor, and retard calculus formation. The zinc elevated concentrations can be sustained for prolonged periods in plaque and saliva following delivery from mouthrinses and toothpastes. Although low concentrations of zinc can both reduce enamel demineralisation and modify remineralisation, the anticariogenic efficacy is yet disputable and not supported by various researches [44].
2. Taste disorders: the role of zinc in taste functions is appreciable at various levels of organization such as taste buds, the taste sense nerve transmission, and brain. Zinc plays an important role in cell structure architecture, maintaining the cell membrane integrity, and functions of various cytoplasmic and membrane enzymes. Early researchers concluded that zinc deficiency secondary to any etiology leads to taste disturbances and thus still zinc depletion is corrected for patients reporting with taste imbalances [45].
3. A study conducted on rodents concluded that zinc-deficient diet can result in parakeratosis of normally orthokeratinized oral mucosa. Hence, zinc deficiency can be a potential risk factor for oral and periodontal diseases. The parakeratotic changes in cheek, tongue, and esophagus are a sign of zinc deficiency. Thickening of the buccal mucosa is a common manifestation along with loss of filiform papillae [46].
4. As stated above, zinc is a cofactor for superoxide dismutase enzyme and various studies have shown lower levels of serum zinc in patients with potentially premalignant disorders like oral leukoplakia. This may be due to consumption of zinc in counter reaction to high content of copper in areca nut or oxidants released during tobacco usage [47].
5. Similarly, concentration of zinc in serum is significantly decreased in oral squamous cell carcinoma and oral submucous fibrosis patients with history of tobacco consumption when compared to the control group and gradually decreased with the duration of the habit. The serum level of zinc was reportedly lower in oral squamous cell carcinoma patients than in oral submucous fibrosis patients [25, 48].
6. As transferrin transports both iron and zinc, the level of zinc increases as the level of iron decreases in iron deficiency patients. Thus, patients of OSMF also suffering from iron deficiency anemia show higher serum levels of zinc [25, 48].
7. Superoxide dismutase which is a natural antioxidant of the body is a Cu-Zn protein complex that has an anticarcinogenic effect in OSMF. Secondly, zinc decreases the activity of copper containing lysyl oxidase enzyme and thus causes inhibition of cross linkage of collagen peptides. It also plays a significant role in promoting collagen degradation through collagenase and matrix metalloproteinase. Zinc thus bears an inverse relationship with copper and thereby interferes with the mucosal absorption of copper. Excess zinc particularly impairs copper absorption as both metals are absorbed through metallothioneins. The ratio of copper to zinc is also believed to be a reliable biomarker in the development and progression towards carcinogenesis [48].
8. On the contrary to the popular belief of protective function of zinc, limited literature suggests carcinogenic effect of zinc [49].

### 3.3. Iron

Iron is the most abundant essential trace element in the human body. The total content of iron in the body is about 3–5 g with most of it in the blood and the rest in the liver, bone marrow, and muscles in the form of heme [50]. Iron is absorbed in the gut from diet in case of depletion and transported in the form of ferritin. Hemosiderin is a golden brown pigment which is a byproduct of metabolism of ferritin and is deposited in the cells of the reticuloendothelial system [51]. Homeostasis of iron maintains the iron levels in serum within normal range only by upregulation or downregulation of absorption mechanism of iron which is unique because it maintains homeostasis by regulating the absorption and never excretion.

#### 3.3.1. Biological Functions

Heme is the major iron containing substance in ferrous or ferric state which is present in hemoglobin, myoglobin, and cytochrome. There are numerous enzymes associated with iron, namely, cytochrome a-c, p450, cytochrome c reductase, catalases, peroxidases, xanthine oxidases, tryptophan pyrrolase, succinate dehydrogenase, glucose-6-phosphate dehydrogenase, and choline dehydrogenase. Heme forms covalent bonds with the globin protein to form hemoglobin which is the major oxygen carrying pigment in RBCs of mammalians. It takes part in a myriad of metabolic cycles such as in the energy producing reactions (the cytochromes of the Krebs cycle) in all the cells and activates the energy producing oxidizing enzymes. Apart from participation in maintaining innumerable physiological and metabolic processes, it is also necessary for DNA, RNA, collagen, antibody synthesis, and so forth [52]. The biological roles of iron in the human body are beyond the scope of this paper and only few important ones have been listed.

#### 3.3.2. Role in Oral Health and Diseases

 The roles of iron in oral health and diseases are summarized as follows:

1. Iron deficiency anemia is the most common manifestation of low serum levels of this important trace element. Microcytic hypochromic RBCs, fatigue, achlorhydria, atrophy of epithelium, loss of attention, irritability, dyspnea, and lowered memory are some of the features of iron deficiency anemia [25]. The oral manifestations of iron deficiency anemia can be summarized as angular cheilitis, atrophic glossitis, generalised oral mucosal atrophy, candidal infections, pallor, and stomatitis. Plummer-Vinson syndrome or Paterson-Kelly syndrome or sideropenic dysphagia is a rare condition characterized by iron deficiency anemia, dysphagia, and koilonychia with women being affected more than men. Dysphagia results from the presence of abnormal esophageal webs which have predisposition towards malignant transformation [53].
2. Oral premalignant lesions and conditions: a significant decrease in serum iron concentrations with elevated total iron-binding capacity has been found in OSMF patients. The decreased iron levels in OSMF patients might be due to utilization of iron in collagen synthesis. In addition, deficient iron in the oral tissues results in decreased vascularity which further facilitates percolation of arecoline (byproduct of areca nut). Further damage is caused by increased arecoline percolation which enhances fibroblastic proliferation and collagen formation [25]. Though majority of the literature suggests that OSMF leads to iron deficiency due to impaired dietary habits, Bhattacharya et al. reported an interesting case where iron deficiency anemia primarily resulted in development of oral submucous fibrosis which was successfully treated by oral administration of iron supplements and antioxidants [54]. Similarly, low serum levels of iron have been assessed in patients suffering from oral leukoplakia.
3. It has also been noted that serum ferritin levels are elevated and serum iron concentrations are decreased with tumor progression in head and neck carcinomas and thus heme can be used as a follow-up tool for patients along with nutritional assessment [47].

### 3.4. Cobalt

The presence of cobalt in animal tissues was first established by Bertrand and Macheboeuf in 1925 which was later confirmed by various researches using spectrographic methods [55, 56]. Cobalt is an essential trace element for the human body and can occur in organic and inorganic forms. In organic form, it forms an integral part of vitamin B12 and has a substantial role in the formation of amino acids and neurotransmitters. Inorganic forms of cobalt are toxic to the human body, and the longer they stay in the body, the more the detrimental effects they cause in cells. Cobalt ions are absorbed within the human body through several pathways: firstly, with food; secondly, by the respiratory system; thirdly, by the skin; and finally, as a component of biomaterials. The cobalt ions enter the body through any of the abovementioned routes and bind with proteins within the bloodstream and get transported with blood to be deposited in tissues and cells. The total body content of cobalt has been estimated between 80 and 300 mcg of vitamin B12 [57–59].

#### 3.4.1. Biological Functions

Vitamin B12, also known as cobalamin, is a water soluble vitamin and contains the biochemically rare element cobalt in the center of a planar tetrapyrrole corrin ring. Vitamin B12 is produced as hydroxocobalamin within bacteria and conversion to methylcobalamin and 5′-deoxyadenosylcobalamin, enzymatically active cofactor forms, occurs within the body. Cyanocobalamin, the fourth vitamer of vitamin B12, can be metabolized in the body to an active coenzyme form and used in food supplements. Erythropoietin, essential for formation of erythrocytes, stimulation is performed by vitamin B12 containing cobalt salts, and thus the deficiency of cobalt is strongly related to disturbances in vitamin B12 synthesis resulting in anemia and hypofunction of thyroid with increased risk of developmental abnormalities and failure in infants [59]. Apart from being an important constituent of these various forms of vitamin B12, the presence of cobalt is necessary for the efficient formation of amino acids and various proteins for myelin sheath generation. Cobalt also plays a decisive role in generating neurotransmitters, which are requisite for proper operation of an organism. On the other hand, excess of cobalt ions within the body might increase the action of thyroid and bone marrow resulting in overproduction of erythrocytes, fibrosis in lungs, and asthma [60].

#### 3.4.2. Role in Oral Health

 The roles of cobalt in oral health are summarized as follows:

1. Cobalt, part of vitamin B12 also referred to as extrinsic factor, is essential for formation of erythrocytes. Thus, the most well-known manifestation of cobalt deficiency in oral cavity is pernicious anemia which is characterized by glossitis, burning sensation, beefy red tongue present in the form of patches or completely red tongue which is also referred to as Hunters' or Moeller's glossitis, and rarely shallow ulcers [61].
2. Apart from erythropoiesis, vitamin B12 also plays a significant role in nerve repair and regeneration. Hence, deficiency of cobalt can have adverse effects such as peripheral neuropathy.
3. Lichen planus and oral lichenoid reactions have been linked to their exposure to Cr, Co, Ni, and amalgam alloys that are released from metal alloys commonly used in dentistry in the oral cavity. These trace metals when released from the metal alloys come into direct contact with oral mucosa, leading to immune mediated damage of basal epithelial keratinocytes and subsequently inducing sensitivity reactions in the form of OLR. Some studies have linked OLR to risk of malignant transformation [62].

### 3.5. Chromium

The word “chrome” is a Greek word which means “color.” Chromium exists in divalent [Cr(II)], trivalent [Cr(III)], and hexavalent [Cr(VI)] oxidation states, with Cr(VI) and Cr(III) being the most stable forms, among which Cr(III) and Cr(VI) are insoluble and soluble forms, respectively. The total body content of chromium is relatively low and is about 0.006 g in an average healthy human adult. Trivalent Cr is an essential trace element and plays an important role in glucose metabolism by serving as a cofactor for insulin action. Hexavalent chromium is a toxic industrial pollutant and has been classified as carcinogen possessing mutagenic and teratogenic properties. Chromium exposure through occupation via inhalation has been associated with various lung, GIT, and central nervous system cancers. Chromium is excreted principally in the urine and faeces and in small quantities in the hair, sweat, and bile [63].

#### 3.5.1. Biological Functions

Chromium is an important trace element for overweight people as it is one of the key minerals in controlling blood sugar and lipid levels. Chromium [Cr(III)] increases the efficacy of insulin and stimulating glucose uptake from the muscles and other tissues being the main ingredient of glucose tolerance factor (GFT). In case of low serum levels of chromium, the circulating level of (GFT) is also less, and, consequently, insulin is less effective in reducing blood sugar. As a result, high blood sugar stimulates further release of ineffective insulin [64, 65]. Chromium is thought to repress p53, a tumor suppressor protein, whose inactivation through mutations is associated with many types of human cancers. Chrome ulcers, corrosive reactions on the nasal septum, acute irritation dermatitis, and allergic eczematous dermatitis have been reported among individuals exposed to hexavalent chromium compounds. Industrial workers exposed to chromates have been documented to be at excessive risk of lung cancer.

As chromium is present in very low amounts in the body, it is difficult to ascertain the deficient state. It is believed that if concentrations of chromium are lower than the normal value of 0.14–0.15 ng/mL in serum, this will indicate the presence of a severe chromium deficiency. In spite of that, elevated plasma levels can coexist with a negative tissue balance. Hyperglycemia may be concomitant with raised plasma chromium and increased urinary excretion. The concentrations of chromium in urine, hair, and body fluids could not reflect the true chromium status of the body [65].

#### 3.5.2. Role in Oral Health and Diseases

The role of chromium in OLR has been discussed earlier [62]. Hyperglycemic status of diabetic patients in undiagnosed chromium deficient state may lead to a wide spectrum of oral manifestations noted in diabetics such as delayed wound healing, suppurative periodontitis, various oral fungal infections, premature periodontal diseases, and hyposalivation [66].

### 3.6. Selenium

Selenium is a vital trace element which is an important component of the antioxidant enzymes such as glutathione peroxides and thioredoxin reductase [67]. The selenium salts required for various cellular functions inside the human body are toxic in excess amounts. Microorganisms reportedly have several selenium containing enzymes, and it is most likely that selenoproteins other than glutathione peroxidase remain to be discovered in higher animals. Historically, endemic in children aged 2–10 years and in women of childbearing age, Keshan disease had a geographic distribution along wide belt-like region throughout mainland China, from northeast to southwest. Typical manifestations were fatigue after even mild exercise, cardiac arrhythmia and palpitations, loss of appetite, cardiac insufficiency, cardiomegaly, and congestive heart failure. The disease was prevalent among people under selenium deficient diet and the patients improved rapidly on fortification. Similarly, selenium-responsive bone and joint disease, Kashin-Beck disease, has also been detected in children aged 5–13 years in China and less so in southeast Siberia. Kashin-Beck disease also occurs in areas with low levels of selenium in cultivation soil.

#### 3.6.1. Biological Functions

Selenium is known to possess immunomodulating and antiproliferative properties and may effect immune response by altering the expression of cytokines and their receptors or making immune cells more resistant to oxidative stress [68, 69]. As a part of enzyme glutathione peroxidase along with vitamin E, catalase, and superoxide dismutase, selenium forms component of one of the imperative antioxidant defence systems of the body. There is also compelling evidence that an unknown selenoenzyme protein has some role in the synthesis of the triiodothyronine hormone from thyroxine [70, 71].

#### 3.6.2. Role in Oral Health and Diseases

 The serum levels of selenium showed progressive reduction from healthy subjects to patients having premalignant lesions like oral leukoplakia and further decrease in patients suffering from oral cancer. There was a reduction in the selenium containing glutathione peroxidase levels as well and there was a concomitant increase in the oxidative stress in the same order [72].

Hence, it is clearly evident that decrease in the concentrations of selenium will result in increased oxidative stress inside the body tissues with inadvertent harmful effects. Thus, nutritional supplementation with trace elements such as selenium is an important rationale in the treatment of premalignant lesions like leukoplakia, conditions as OSMF, and oral cancer patients to reduce oxidative stress inside the body [54].

A recent study evaluated the anti-inflammatory and antioxidant effect of selenium by administration in patients suffering from oral mucositis secondary to high dose chemotherapy. The researchers maintained that adequate supplementation of selenium could produce cytoprotective effects and antiulcer activity and concluded that selenium can effectively reduce the duration and severity of oral mucositis in these patients [73].

### 3.7. Molybdenum

Molybdenum minerals have been known throughout history, but the element was discovered by Carl Wilhelm Scheele in 1778 and first isolated in 1781 by Peter Jacob Hjelm.

#### 3.7.1. Biological Functions

Molybdenum, as a component of molybdoprotein, takes part in the formation of active sites for various enzymes. The three principal molybdenum-containing enzymes are xanthine dehydrogenase/oxidase, aldehyde oxidase, and sulphite oxidase. A molybdenum-containing enzyme has some role to play in purine catabolism. It also influences protein synthesis and growth of the body [74]. Molybdenum has an antagonistic effect against copper; thus, high concentrations of molybdenum can reduce copper absorption and subsequently lead to copper deficiency [75].

#### 3.7.2. Role in Oral Health and Diseases

 Boron, vanadium, and molybdenum are believed to possess a cariostatic effect. Various researches from Hungary and New Zealand strongly suggested molybdenum-flouride interaction to have a strong cariostatic effect. However, the cariostatic effect of molybdenum has been critically reviewed in the literature with inconclusive results. Nonetheless, teeth enamel accrues considerable amounts of molybdenum. Further researches or investigations are warranted to derive reliable observations [76].

### 3.8. Fluorine

Fluorine makes negligible part of body weight and enters the system principally through drinking water and to a lesser extent through foods.

#### 3.8.1. Biological Functions

Fluorine, in the form of fluorapatite crystals, is an important part of the organized matrix of hard tissues like bone and teeth. It is also believed that fluoride, in combination with calcium, stimulates osteoblastic activity [64].

#### 3.8.2. Role in Oral Health and Diseases

Low levels of fluoride in drinking water have been associated with dental decay. The excessive concentrations of fluoride during calcification stage of the teeth can result in a kind of enamel hypoplasia termed dental fluorosis. Clinically, dental fluorosis can vary from small white opacities in the enamel to severe mottling of the tooth structure with increasing severity. The overall effect of excessive fluoride intake on the dental structure depends on many factors such as concentration of fluoride in drinking water, stage of calcification of teeth when exposure occurred, duration of exposure, and amount of exposure [61].

### 3.9. Iodine

Iodine is a vital trace element required at all stages of life especially during formative years. It is important to sustain the daily functions of human body and deficiency or excess can have significant adverse effects on the body.

#### 3.9.1. Biological Functions

 Iodine is an essential component of thyroid hormones, that is, tetraiodothyronine (T4 or thyroxine) and triiodothyronine (T3). It plays a significant role in the functioning of the parathyroid glands. Iodine plays an important role in general growth and development of the body along with maintaining metabolic processes [64].

#### 3.9.2. Role in Oral Health and Diseases

There can be innumerable symptoms of iodine deficiency or excess. The deficiency of iodine is more commonly evident. Most commonly reported symptoms of iodine deficiency are extreme fatigue, irritability, mental disturbances, weight gain, facial puffiness, constipation, and lethargy. Untreated infants have the risk of developing cretinism and end up suffering from poor growth and mental retardation [64].

It has also been hypothesized that dietary deficiency or excess of iodine plays an imperative role in oral mucosa and in salivary glands physiology. Salivary glands can protect their own cells from peroxidation due to iodine concentrating ability through sodium iodide symporter and peroxidase activity. Iodide seems to have a primitive antioxidant function in iodide concentrating organisms. Significant role of iodine in oral immune defense mechanism may be substantiated by high concentration of iodine in thymus. S. Venturi and M. Venturi also suggested that these actions of iodides might be important for prevention of various oral and salivary glands diseases [77].

It was also evident in a study conducted by Littleton and Frohlich in 1993 that the skeletal remains from iodine rich zones of the world showed greater attrition, lesser dental caries, and reduced premature teeth loss. Early loss of teeth can be a primary factor in undernutrition and loss of health and compromised quality of life [78].

Deficiency of iodine is not uncommon in various parts of the world population. Worldwide fortification of edible salt has been undertaken to offset the iodine deficiency. Hypothyroidism is characterized by decreased levels of thyroid hormone. With respect to oral involvement, there can be evident thickening of lips due to deposition of glycosaminoglycans in the subcutaneous tissues. Similarly, macroglossia of tongue can be seen because of the same reason. If children are affected, there may be delayed eruption of teeth without any influence on teeth formation [53].

Hyperthyroidism in adults can result in diffuse brown pigmentation of the gingiva, buccal mucosa, palate, and tongue similar to Addison's disease. The mechanism through which the stimulation of melanin synthesis occurs is yet unclear but pigmentation tends to resolve with the treatment of thyroid abnormality [79].

## 4. Detection of Trace Elements and Assessment of Nutritional Status

This was done as follows:

1. Though various methods have been employed to determine the presence of trace elements, it is a cumbersome and nonfruitful job due to their wide distribution within the living tissues and enzyme systems. Colorimetric and spectrographic methods are used commonly to analyse the amount of trace elements. Typically, spectroscopy and electrochemical methods are preferred for solitary element analysis whereas neutron activation analysis and spectroscopic methods are used for determination of more than one element [11].
2. The most easily determined deficiency is of iron which can be determined by performing laboratory tests [80]. Bone marrow smear containing no stainable iron is definitive. Elevated total iron-binding capacity, low serum iron level, and a low serum ferritin concentration are considered diagnostic for iron deficiency. Recently, newer approaches like erythrocyte zinc porphyrin assay have also been used in primary screening tests for assessing iron status [81].
3. The reported optimal plasma or serum ratio between copper and zinc is 0.70–1.00. Diagnosing zinc deficiency is a persistent challenge as mentioned before in the paper. Plasma or serum zinc levels are the most commonly used indices for evaluating zinc deficiency. Severe Cu deficiency can be found by testing for low plasma or serum copper levels, low ceruloplasmin, and low superoxide dismutase levels but these are not very sensitive tests and fail to determine marginal copper deficiency [16, 82].
4. The assessment of the iodine nutritional status of a population or group living in an area or region suspected to be iodine-deficient area can be performed by assessment of the goitre rate, measurement of urinary iodine excretion, and determination of the level of blood T3, T4, or TSH.
5. Tissue chromium stores apparently do not truly reflect the blood chromium; thus, serum chromium concentration is not a good indicator of chromium status. It has been postulated that serum chromium levels lower than 0.14–0.15 ng/mL indicate the presence of a severe chromium deficiency. Excessive exposure of individual to chromium via occupation or accident may be reflected by elevated serum chromium.
6. Different tissues such as blood, hair, and nails have been analysed for determining the nutritional status of selenium. Generally, these tissues can provide a sound appraisal of selenium status if dietary selenium intake is relatively uniform.
7. Tissue levels status of other trace elements in normal individuals is difficult to determine.

## 5. Conclusion

It is one of the most difficult tasks to diagnose trace element deficiencies nutritionally as well as clinically. The deficient intake of an essential trace element may diminish significant biological functions within tissues and restoration of physiological levels of that element relieves the impaired function or prevents impairment. The human body has an elaborate system for managing and regulating the amount of key trace metals circulating in blood and stored in cells. The abnormal levels of these trace elements may develop when the body fails to function properly or there are improper levels in dietary sources. There are convincing lines of evidence that a diet rich in antioxidants and essential minerals is indispensable for a healthy mind and body. Preventive medicine in the recent years has gained more attention than anything else as quoted aptly, “prevention is better than cure.” Selective reproduction of association between preventive medicine and various trace elements has been presented in Table 2 [5]. Oral and general health cannot be viewed independently and as a matter of fact the oral cavity can mirror the systemic health effectively. The combination of various micronutrients and trace elements has been used as a treatment strategy for oral diseases like oral leukoplakia, oral submucous fibrosis, oral cancer, and so forth as their collective outcome is more beneficial as compared to solitary application. Hence, knowledge of the clinical aspects of trace elements is becoming requisite for general as well as oral physicians.

## Figures and Tables

**Table 1:** Showing trace elements, their RDI, RDA, and UL in the body, and rich dietary sources [64, 66, 83].

Trace element	Recommended daily intake (RDI)	Recommended dietary allowance (RDA)	Tolerable upper intake level (UL)	Dietary sources
Copper	2000 *μ*g	Children 1 to 3 years old: 340 mcg/day; 4 to 8 years old: 440 mcg/day; 9 to 13 years old: 700 mcg/day; 14 to 18 years old: 890 mcg/day Men and women aged 19 years and older: 900 mcg/day Pregnancy: 1000 mcg/day Lactation: 1300 mcg/day	Children 1 to 3 years old: 1 mg/day; 4 to 8 years old: 3 mg/day; 9 to 13 years old: 5 mg/day; 14 to 18 years old: 8 mg/day Adults 19 years old and above (including lactation): 10 mg/day Pregnancy: 8 mg/day	Oysters, other shell fish, whole grains, beans, nuts, potatoes, organ meats (kidney, liver), dark leafy greens, dried fruits, and yeast

Iron	18 mg	Children 1 to 3 years old: 7 mg/day; 4 to 8 years old: 10 mg/day; 9 to 13 years old: 8 mg/day Boys 14 to 18 years old: 11 mg/day Girls 14 to 18 years old: 15 mg/day Adults: 8 mg/day for men aged 19 and older and women aged 51 and older Women 19 to 50 years old: 18 mg/day Pregnant women: 27 mg/day Lactating mothers: 10 mg/day	Infants and children from birth to the age of 13: 40 mg/day Children aged 14 and adults (including pregnancy and lactation): 45 mg/day	Haem iron: liver, meat, poultry, and fish Nonhaem iron: cereals, green leafy vegetables, legumes, nuts, oilseeds, jaggery, and dried fruits

Zinc	15 mg	Infants and children 7 months old to 3 years old: 3 mg/day; 4 to 8 years old: 5 mg/day; 9 to 13 years old: 8 mg/day Girls 14 to 18 years old: 9 mg/day Boys and men aged 14 and older: 11 mg/day Women 19 years old and above: 8 mg/day Pregnant women: 11 mg/day Lactating women: 12 mg/day	Infants: 4-5 mg/day Children 1 to 3 years old: 7 mg/day; 4 to 8 years old: 12 mg/day; 9 to 13 years old: 23 mg/day; 14 to 18 years old: 34 mg/day Adults 19 years old and above (including pregnancy and lactation): 40 mg/day	Animal food: meat, milk, and fish Bioavailability of zinc in vegetable food is low

Cobalt	6 *μ*g	Infants: 0.5 mcg Children 1–3 years old: 0.9 mcg; 4–8 years old: 1.2 mcg; 9–13 years old: 1.8 mcg Older children and adults: 2.4 mcg Pregnant women: 2.6 mcg Lactating mothers: 2.8 mcg	Not known	Fish, nuts, green leafy vegetables (broccoli, spinach), cereals, and oats

Chromium	120 *μ*g	Children 1 to 3 years old: 11 mcg; 4 to 8 years old: 15 mcg Boys 9 to 13 years old: 25 mcg Men 14 to 50 years old: 35 mcg Men 51 years old and above: 30 mcg Girls 9 to 13 years old: 21 mcg; 14 to 18 years old: 24 mcg Women 19 to 50 years old: 25 mcg; 51 years old and above: 20 mcg Pregnant women: 30 mcg Lactating women: 45 mcg	Doses larger than 200 mcg are toxic	Best sources: processed meats, whole grains, and spices

Molybdenum	75 *μ*g	Children 1 to 3 years old: 17 mcg/day; 4 to 8 years old: 22 mcg/day; 9 to 13 years old: 34 mcg/day; 14 to 18 years old: 43 mcg/day Men and women aged 19 years and above: 45 mcg/day Pregnancy and lactation: 50 mcg/day	Children: 300–600 mcg/day Adults (including pregnancy and lactation): 1100–2000 mcg/day	Animal food: liver; vegetables: lentils, dried peas, kidney beans, soybeans, oats, and barley

Selenium	70 *μ*g	Children 1–3 years old: 20 micrograms/day Children 4–8 years old: 30 micrograms/day Children 9–13 years old: 40 micrograms/day Adults and children 14 years old and above: 55 micrograms/day Pregnant women: 60 micrograms/day Breastfeeding women: 70 micrograms/day	The safe upper limit for selenium is 400 micrograms a day in adults	Liver, kidney, seafood, muscle meat, cereal, cereal products, dairy products, fruits, and vegetables

Iodine	150 *μ*g	Children 1 to 8 years old: 90 mcg/day; 9 to 13 years old: 120 mcg/day Children aged 14 and adults: 150 mcg/day Pregnant women: 209 mcg/day Lactating mothers: 290 mcg/day	Children 1 to 3 years old: 200 mcg/day; 4 to 8 years old: 300 mcg/day; 9 to 13 years old: 600 mcg/day; 14 to 18 years old: 900 mcg/day Adults above the age of 19 including pregnant and breastfeeding women: 1100 mcg/day	Best sources: seafoods (sea fish and sea salt) and cod liver oil Small amounts: milk, vegetables, and cereals

Fluorine	In drinking water: 0.5 to 0.8 mg	Children 1 through 3 years old: 0.7 mg; 4 to 8 years old: 1 mg; 9 to 13 years old: 2 mg; 14 to 18 years old: 3 mg Men 19 years old and above: 4 mg Women 14 years old and above (including pregnant or breastfeeding women): 3 mg	0.7–9 mg for infants 1.3 mg for children 1 to 3 years of age 2.2 mg for children 4 to 8 years of age 10 mg for children above 8 years old, adults, and pregnant and breastfeeding women	Drinking water, foods (sea fish and cheese), and tea

**Table 2:** Trace elements and preventive medicine [5].

Prevention	Trace element involved
Predisposition to anemia	Iron, cobalt, copper
Reduction in antioxidant potential	Zinc, iron, manganese, selenium, copper
Promotion of aging and its cause	Zinc, copper, selenium, chromium
Immunodeficiency	Zinc, iron, copper, selenium
Increased carcinogenicity	Zinc, copper, selenium
Promoted atherosclerosis	Zinc, selenium, iron, copper, chromium
Increased incidence of diabetes mellitus	Chromium, zinc, selenium
Predisposition to taste disorder	Zinc
Predisposition to dental caries	Fluorine, molybdenum?
Predisposition to goiter	Iodine
